# Efficient removal of arsenic (V) and methyl orange from aqueous solution using hollow magnetic chitosan composite microspheres: Low arsenic concentration, high adsorption capacity, and minimal adsorbent requirement

**DOI:** 10.1371/journal.pone.0337694

**Published:** 2025-12-12

**Authors:** Zahra Hashemi Jorshahri, Ali Akbari

**Affiliations:** Chemical Engineering Faculty, Sahand University of Technology, Tabriz, Iran; Adama Science and Technology University, ETHIOPIA

## Abstract

In this study, hollow Fe_3_O_4_-SiO_2_-chitosan adsorbent with an optimal chitosan concentration of 2% (w/v) was synthesized to enhance arsenic (V) adsorption performance from low-concentration aqueous solutions. The hollowing process was used to enhance the surface area of the adsorbent and compensate for the surface area reduction of Fe_3_O_4_ nanoparticles induced by SiO_2_ and chitosan coating layers. Furthermore, the adsorption properties of organic pollutants were evaluated using methyl orange as the adsorbate. The microspheres underwent systematic characterization, and their arsenic (V) and methyl orange adsorption capacities were evaluated under various influencing factors. The results indicated improved surface area (202.174 m^2^/g) compared to non-hollow magnetic adsorbents (110 m^2^/g) reported in previous studies. Complete arsenic (V) removal (100%) was achieved within 60 minutes at a concentration of 0.2 mg/L, using an adsorbent dose of 0.012 g at pH 5. The optimal adsorbent doses for methyl orange (0.1 g/L) and arsenic (V) (0.5 g/L) were notably lower than those reported in previous studies. The electrostatic attraction was likely the dominant mechanism for arsenic (V) adsorption, whereas methyl orange adsorption may involve n-π interactions, hydrogen bonding, and electrostatic forces. The adsorption process followed the pseudo-second-order kinetics model and the Langmuir isotherm, with maximum adsorption capacities of 175.086 mg/g for arsenic (V) at pH 5 and 2399.910 mg/g for methyl orange at pH 3. The adsorbent showed significant potential for removing arsenic (V) and methyl orange, particularly from acidic wastewater. Moreover, the adsorbent maintained significant portion of its initial adsorption capacity for As(V) and methyl orange even in the presence of competing anions such as phosphate, sulfate, chloride, and nitrate. After four adsorption–desorption cycles, it retained over 90% of its adsorption capacity, demonstrating excellent selectivity, stability, and strong potential for the effective removal of both As(V) and methyl orange from aqueous solutions.

## 1. Introduction

The pollution of water resources by hazardous inorganic and organic pollutants has emerged as a significant global problem, negatively impacting aquatic ecosystems and public health [1–3]. This environmental crisis has driven scientists to develop novel, economical, and efficient water treatment techniques and materials [4,5]. Among the most dangerous pollutants in water systems are heavy metals and organic dyes, which pose serious threats to human and environmental health [6,7]. Examples of toxic heavy metals include copper (Cu), lead (Pb), arsenic (As), zinc (Zn), nickel (Ni), chromium (Cr), mercury (Hg), and cadmium (Cd) [8]. In addition, dyes are categorized based on their chemical structure and water solubility into three groups: cationic dyes like methyl blue and malachite green, anionic dyes such as congo red and methyl orange, and non-ionic dyes like dispersion orange 37 [9]. Wastewater from various industrial activities often contains these hazardous pollutants, which can enter the environment through wastewater discharges. The resist degradation and accumulation of heavy metals and dyes in bodies of living organisms lead to various health issues and disruptions [10]. Arsenic, a harmful metalloid element, is considered one of the most dangerous contaminants for human health [11,12]. Inorganic arsenic primarily exists in two forms: trivalent arsenite (As(III)) and pentavalent arsenate (As(V)). In aqueous solutions, As(III) can be present as H_3_AsO_3_, H_2_AsO_3_ ⁻ , HAsO_3_^2^ ⁻ , or AsO_3_^3^ ⁻ depending on the pH, while As(V) can exist as H_3_AsO_4_, H_2_AsO_4_ ⁻ , HAsO_4_^2^ ⁻ , or AsO_4_^3^⁻ [13,14]. Major sources of arsenic contamination in water include both natural processes, such as rock weathering and volcanic emissions, and human activities, including industrial waste, mining, and agricultural practices [13]. Arsenic is used in semiconductors and LEDs, and its presence is essential for industrial growth; however, rapid industrialization has increased its levels in the environment, often exceeding safe limits [15].Due to its significant toxicity, the World Health Organization (WHO) and the United States Environmental Protection Agency (USEPA) reduced the permissible limit of arsenic in drinking water from 50 to 10 μg/L in 1993 [16]. Even at low concentrations, arsenic can pose significant risks to humans, aquatic organisms, terrestrial animals, and plants [17,18]. Long-term exposure to arsenic can lead to skin, liver, kidney, and lung cancer, as well as cardiovascular diseases, gastrointestinal disorders, and neurological damage [13,19]. Methyl orange (MO), a synthetic anionic azo dye, is another major pollutant widely found in wastewater from industries such as pharmaceuticals, printing, food, paper, textiles, and research laboratories [20]. MO wastewater contains complex organic compounds that quickly show color in the water [21]. MO poses significant health risks, causing skin and eye irritation, respiratory problems, and, in severe cases, symptoms like vomiting, diarrhea, and even death [20,22]. Therefore, it is crucial to develop effective treatment methods to remove such pollutants from wastewater before discharge into the environment. The main water treatment methods for removing heavy metals and dyes include adsorption, ion exchange, electrochemical processes, membrane filtration, chemical precipitation, biodegradation, electrodialysis precipitation, photocatalysis, and oxidation [23–25]. Among these, adsorption is particularly favored due to its low operating costs, simple and diverse design, and high efficiency especially the use of nanoparticles as adsorbents [26–28]. However, the effectiveness of adsorption heavily relies on the properties of the adsorbent, which should exhibit high surface area, stability, and capacity for pollutant adsorption [9]. Research on wastewater treatment using the adsorption method has focused on the development of available, cost-effective, efficient, easy-to-process, reusable, and environmentally friendly adsorbents [29,30]. Recently, bio-based materials have gained increasing attention in wastewater treatment due to their biodegradability, abundance, cost-effectiveness, and environmental compatibility. These materials provide a sustainable alternative to conventional synthetic adsorbents. Among them, waste biomass from agricultural and industrial sources has been widely explored as a renewable feedstock for preparing efficient adsorbents for water purification [31]. In addition, porous activated carbon structures derived from biopolymers or agricultural residues have demonstrated excellent performance in removing organic pollutants and heavy metals due to their large surface area and tunable pore structure [32]. Furthermore, chitosan-based derivatives and biochar materials produced from agricultural by-products have also shown promising adsorption capabilities toward various pollutants [33]. Chitosan, a natural biopolymer, has shown great promise in this regard due to its functional groups, such as amino (-NH_2_) and hydroxyl (-OH) groups, which provide active sites for pollutant adsorption. Chitosan is hydrophilic, biodegradable, and cost-effective, making it an attractive option for removing heavy metals and dyes from water [29,34,35]. For example, Sakkayawong et al. [36] reported that the maximum adsorption capacities of a synthetic dye by chitosan were 68–156 mg/g at pH = 11 and 20–60°C, respectively. Despite its advantages, the use of unmodified chitosan as an adsorbent is limited by its low porosity, surface area, and stability, particularly in acidic environments. To overcome these limitations, various modification techniques have been explored [8,37,38]. One common method is crosslinking, which enhances the mechanical strength and stability of chitosan, thereby improving its adsorption efficiency. Crosslinking agents such as formaldehyde, glutaraldehyde, and epichlorohydrin are frequently used for this purpose [13]. Another challenge in adsorption processes is the separation of adsorbents from the treated water, which, if not properly addressed, can negatively affect the aquatic ecosystem. Traditional separation methods, such as filtration and centrifugation, are costly and time-consuming [39]. In contrast, magnetic separation utilizes the magnetic properties of nanoparticles combined with an external magnetic field, providing a faster and more cost-effective solution [40]. Fe_3_O_4_ is a magnetic material widely used in wastewater treatment due to its temperature stability, biocompatibility, low cost, small particle size, and high surface area [41,42]. Men et al. [43] synthesized a poly sodium 4-styrene sulfonate grafted magnetic chitosan microspheres to remove methylene blue from water. The adsorbent showed an adsorption capacity of 989 mg/g for methylene blue at 25°C, and the microspheres were efficiently separated using a magnet, which facilitated reusability. However, Fe^2+^ ions in Fe_3_O_4_ can easily oxidize to Fe^3+^ ions, which are unstable and tend to aggregate. This issue can be addressed by coating the nanoparticles with a protective shell, such as silica (SiO_2_), which stabilizes them and prevents dissolution at low pH [39,44]. However, successive coating layers often reduce the surface area of Fe_3_O_4_ nanoparticles, leading to a decrease in adsorption capacity. For example, Tabaraki et al. [45] synthesized arginine-modified magnetic Fe_3_O_4_/chitosan nanoparticles for the biosorption of multiple dyes, including Titan Yellow, Fuchsine Acid, and Indigo Carmine, in a ternary mixture. They observed a significant reduction in surface area and pore volume after incorporating chitosan and arginine with Fe_3_O_4_ nanoparticles. Similar results were reported by Khan et al. [46] and Helmi et al. [47]. Thus, developing magnetic composites that retain strong magnetic properties and high surface area despite coating layers is essential. At low pollutant concentrations, the interactions between pollutants and adsorbents are less frequent, making effective adsorption more challenging. One of the main challenges in arsenic removal is achieving effective adsorption at low concentrations. Song et al. [48] prepared magnetic composite microparticles based on chitosan derivatives and achieved nearly 100% arsenic (V) removal at a concentration of 0.5 mg/L with an adsorbent dose of 5 g/L Under 40°C. Rawat et al. [49] synthesized iron oxyhydroxide chitosan beads for As(V) removal in the 0.24–15.64 mg/L concentration range. They achieved over 90% removal efficiency at 0.24 mg/L concentration with an adsorbent dose of 1.2 g/L. Despite notable progress in the chitosan-based adsorbents for low-concentration arsenic removal, most studies focus on maximizing removal efficiency and require high adsorbent doses. There is limited research on developing adsorbents that ensure both high efficiency and process economics without the need for high adsorbent doses. These challenges are crucial because magnetic adsorbents with reduced surface areas or high-dose requirements significantly impair the operational and economic viability of the adsorption process. In this study, hollow Fe_3_O_4_-SiO_2_-chitosan microspheres were synthesized to achieve a high surface area while maintaining the magnetic properties of Fe_3_O_4_ nanoparticles despite the SiO_2_ and chitosan coatings. The synthesis process began with the preparation of polystyrene (PS) microspheres, and their surface was coated with Fe_3_O_4_ nanoparticles. The outer layer of these PS-Fe_3_O_4_ microspheres was coated with SiO_2_. To create a hollow structure, a high-temperature calcination process was employed. Finally, the optimal chitosan concentration for effective As(V) removal at low concentrations was evaluated and applied to the synthesis process. It is necessary to explain that insufficient chitosan results in a lack of adsorption sites, while excessive chitosan leads to molecule accumulation on the surface, limiting pollutant access to the hollow structure. The optimal chitosan concentration maximizes the active sites of chitosan without surface saturation, enhancing chemical interactions with contaminants and improving arsenic adsorption at low concentrations. Given the increasing As(V) concentrations in drinking water in the Azerbaijan region, this study aimed to remove As(V) ions from polluted water effectively. MO dye was also used as an anionic organic pollutant to evaluate adsorbent performance for organic pollutants. The study focuses primarily on arsenic removal at low concentrations while also investigating a broader concentration range (0.2–62 mg/L) to analyze adsorption behavior and evaluate factors such as pH, adsorbent dose, and adsorption isotherms and kinetics. In addition, various analytical techniques, including vibrating sample magnetometer (VSM), transmission electron microscopy (TEM), X-ray diffraction (XRD), Fourier transform infrared spectroscopy (FT-IR), Field emission scanning electron microscopy (FESEM), and N_2_ adsorption-desorption analysis were used to characterize the synthesized composites.

## 2. Experimental

### 2.1. Materials

Iron (III) chloride hexahydrate (FeCl_3_.6H_2_O), Iron (II) chloride tetrahydrate (FeCl_2_·4H_2_O), tetraethyl orthosilicate (TEOS), methyl orange (MO, C_14_H_14_N_3_NaO_3_S), sodium hydroxide (NaOH), and acetic acid (CH_3_CO_2_H) were purchased from Merck (Darmstadt, Germany). Chitosan (medium molecular weight, DDA ∼ 90%), styrene (St), methacrylic acid (MAA), glutaraldehyde (GA,50% w/w), ammonia solution (NH_3_.H_2_O, 28% w/w), hydrochloric acid (HCl, 37%), ammonium persulfate (APS), and sodium arsenate heptahydrate (Na_2_HAsO_4_.7H_2_O), Na_2_HPO_4_, Na_2_SO_4_, NaCl, and NaNO_3_ were acquired from Sigma-Aldrich (St. Louis, MO, USA). Ethanol (99.98%) was obtained from Zanjan Kimia Alcohol Co. Zanjan, Iran.

### 2.2. Synthesis of carboxyl-modified PS microspheres

The carboxyl-modified polystyrene (PS) microspheres were synthesized through emulsifier-free MAA and St monomer polymerization [50]. Before use, St and MAA were purified by vacuum distillation to remove inhibitors. First, 95 mL deionized water was added to a three-necked round-bottom flask, and then, oxygen was removed using room-temperature N_2_ purging. Next, 10 mL St and 2 mL MAA were added to the flask and stirred in the presence of N_2_ for 15 min. 0.05 of APS was dissolved in 5 mL deionized water and added to the mixture. The reaction continued for 24 h at 72°C. The product was washed with deionized water and ethanol for three times and collected through centrifugation. The carboxyl-modified PS, in the form of fine white powder, was obtained after drying it in a vacuum oven at 50°C.

### 2.3. Synthesis of magnetic carboxyl-modified PS microspheres (PS-Fe_3_O_4_)

The Fe_3_O_4_ nanoparticles were grafted to the carboxyl-modified PS microspheres using the coprecipitation method to create magnetic carboxyl-modified PS microspheres [51]. An ultrasonic bath was used to disperse 2 g of PS microspheres in 100 mL deionized water, and the mixture was cooled in an ice-water bath under nitrogen gas bubbling. Then, a solution containing 0.8 g FeCl_3_.6H_2_O and 0.53 g FeCl_2_.4H_2_O dissolved in 20 mL deionized water was added to the PS and deionized water mixture under stirring. A mixture with a light-yellow color was created. After 1 h reaction in an ice bath, the flask was transferred to an oil bath at 85°C, forming a dark yellow mixture. The mixture was subjected to the gradual dropwise addition of 25 mL NH_3_ ⋅ H_2_O, leading to a progressive change in color to black. The mixture was cooled to room temperature after 1 h stirring at 85°C. After several times washing with water and ethanol; the resulting magnetic PS microspheres were centrifuged and dried in a vacuum oven.

### 2.4. Synthesis of hollow Fe_3_O_4_-SiO_2_ microspheres

200 mg of synthesized magnetic PS microspheres was mixed with 12 mL deionized water and 170 mL ethanol and rapidly stirred for 40 min. Subsequently, 4 mL NH_3_·H_2_O was added to the mixture. Then, a mixture containing 1200 μL (1.2 mL) TEOS and 10 mL ethanol was prepared and gradually added drop by drop into the initial mixture. After adding drops, the mixture was continually stirred for 8 h. The final product was washed with ethanol and deionized water three times and dried in a vacuum oven at 50°C for 12 h. Ultimately, the final microspheres were calcinated for 7 h at 500°C to achieve the hollow Fe_3_O_4_-SiO_2_.

### 2.5. Synthesis of hollow Fe_3_O_4_-SiO_2_ with different chitosan concentration

**Fig 1:**
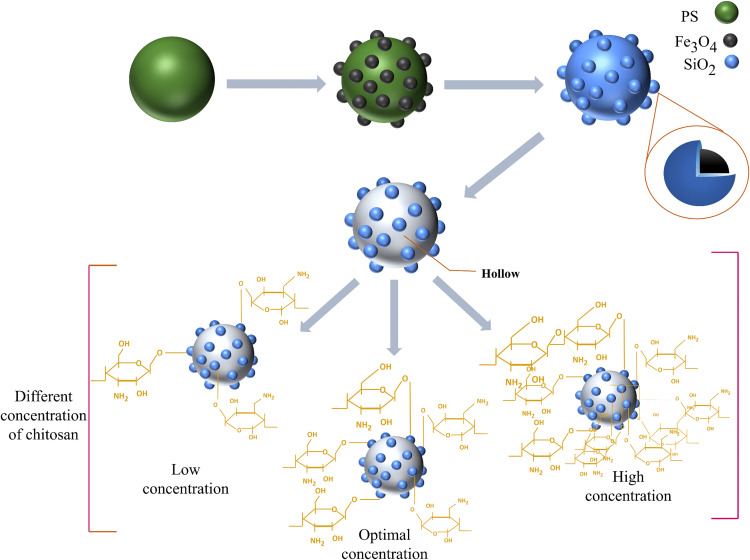
Synthesis steps of hollow Fe_3_O_4_-SiO_2_-chitosan with different concentrations of chitosan.

Four different chitosan solutions were prepared, containing 1, 1.5, 2, and 2.5 g chitosan in 100 ml of 2.0% (v/v) acetic acid solution. Then, 1 g hollow Fe_3_O_4_-SiO_2_ microspheres were dispersed in 100 mL deionized water and were added dropwise using a glass syringe into the chitosan solution under vigorous stirring. The mixtures were stirred consistently at room temperature for 180 min. Subsequently, the obtained products were washed three times with deionized water to remove residual unreacted chitosan. Then, the synthesized composites with four different concentrations of chitosan were dispersed in 100, 200, 300, and 400 mL of deionized water, respectively. The hollow Fe_3_O_4_-SiO_2_-chitosan microspheres were obtained by crosslinking with GA (the ratio of GA to chitosan was 1:2 (w/w)) and gently stirring for 2 hours in a water bath at 40°C. Fig 1 illustrates the various stages of the synthesis process. The optimal concentration of chitosan (w/v%) was determined based on its most suitable value in the adsorbent structure without surface saturation.

### 2.6. Characterization

XRD (ULTIMA. IV, RIGAKU Co., Japan) was performed over the 10° < 2θ < 80° range using Cu-Kα (λ = 1.54 Å) radiation to evaluate the structure and crystallinity of the prepared materials. FT-IR spectra of samples were acquired at 4000–500 cm^− 1^ wave number range using a Tensor 27 spectrometer (BRUKER, Germany). The morphology and structure of the samples were assessed through the use of Field Emission Scanning Electron Microscopy (FE-SEM, SIGMA VP, ZEISS, Germany) and Transmission Electron Microscopy (TEM10, ZEISS, Germany). The Micromeritics BET Analyzer (USA) was utilized to measure the surface area and pore size of the synthesized microspheres. The magnetic features of PS-Fe_3_O_4_, PS-Fe_3_O_4_-SiO_2_, hollow Fe_3_O_4_-SiO_2_, and hollow Fe_3_O_4_-SiO_2_-chitosan microspheres were evaluated using a value stream map (VSM) (MDKB, Iran) under ambient temperature conditions.

### 2.7. Adsorption experiments

Stock solutions of As(V) and MO (1000 mg/L) were prepared by adding the appropriate quantity of sodium arsenate heptahydrate (Na_2_HAsO_4_.7H_2_O) and MO (C_14_H_14_N_3_NaO_3_S) in 1000 mL of deionized water. The stock solutions were gradually diluted to obtain standard solutions with As(V) concentrations ranging from 0.2 to 62 mg/L and MO solution concentrations from 35 to 300 mg/L. The influence of pH value (2–8), adsorbent dose (0.05 to 2 g/L), initial concentration (0.2 to 62 mg/L for As(V) and 35–300 mg/L for MO), and contact time (5–200 min), temperature (25,35,45˚C) for As(V) and MO adsorption were evaluated. Co-existing ion effects were investigated using NaCl and NaNO_3_ (50,100,150, and 200 mg/L) for competition with MO and Na_2_HPO_4_ and Na_2_SO_4_ (5, 10, 20, 30, and 50 mg/L) for competition with As(V). In summary, 12 and 20 mg of hollow Fe_3_O_4_-SiO_2_-chitosan microspheres were added to 25 mL of As(V) and 200 mL of MO solutions, respectively. 0.1M HCl and 0.1M NaOH solutions were used for pH adjustment. The solutions were then stirred for 60 minutes at room temperature using a mechanical shaker. After stirring, a small magnet was used to extract the adsorbent from the solution. During the reusability test, the adsorbent was regenerated using 0.2 M NaOH + 30% ethanol for MO and 0.1 M NaOH solutions for As(V) for 24 hours, with the regeneration solutions refreshed every 12 hours [13,52]. Four successive adsorption–desorption cycles were conducted, and the adsorption capacities of As(V) and MO were calculated for each cycle. The arsenic concentration was measured using an Analyst 200 Atomic Absorption Spectrophotometer (Perkin Elmer), while the MO concentration was determined using a UV-1800 spectrophotometer (Shimazu, Japan) at a wavelength of 464 nm. The changes of As(V) and MO concentrations in the solution before and after the adsorption experiment were used to calculate the adsorption capacity q_e_ (mg/g) and removal efficiency (%R). q_e_ and %R of the hollow Fe_3_O_4_-SiO_2_-chitosan were calculated using Eq. (1) and Eq. (2), respectively [53].


$$q_{e}=\frac{\left(C_{i}-C_{e}\right)}{m}\times \;V$$ (1)

$$\%R=\frac{\left(C_{i}-C_{e}\right)}{C_{i}}\;\times \text{1}\text{0}\text{0}$$ (2)

Where q_e_ (mg/g) denotes the adsorption capacity at equilibrium, C_i_ (mg/L) and C_e_ (mg/L) show the initial and equilibrium concentrations of the As(V) or MO solution, respectively. V (L) represents the volume of As(V) or MO solutions, and m (g) refers to the weight of the adsorbent. All adsorption experiments were replicated at least three times and an average of values was reported.

## 3. Results and discussion

### 3.1. Characterization of adsorbent

**Fig 2:**
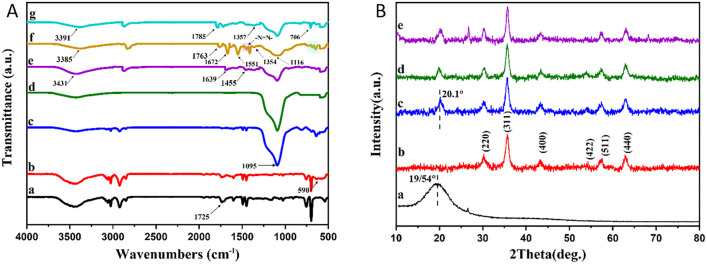
(A) FTIR spectra of (a) carboxyl-modified PS, (b) PS-Fe_3_O_4_, (c) PS-Fe_3_O_4_-SiO_2_, (d) hollow Fe_3_O_4_-SiO_2_, (e) hollow Fe_3_O_4_-SiO_2_-chitosan, (f) hollow Fe_3_O_4_-SiO_2_-chitosan-MO, (g) hollow Fe_3_O_4_-SiO_2_-chitosan-As(V) microspheres. **(B)** XRD patterns of (a) carboxyl-modified PS, **(b)** PS-Fe_3_O_4_, **(c)** PS-Fe_3_O_4_-SiO_2_, (d) hollow Fe_3_O_4_-SiO_2_, (e) hollow Fe_3_O_4_-SiO_2_-chitosan, microspheres.

Fig 2A shows the various FTIR spectra of the adsorbent both during the preparation stages and after the adsorption of pollutants. In spectrum a, two peaks at 1492 cm^-1^ and 1656 cm^-1^ were associated with the aromatic groups in the PS. Furthermore, the peaks at 2852 cm^-1^ and 2923 cm^-1^ indicated the stretching vibrations of the aliphatic C-H bonds. There was also a peak at 1725 cm^-1^, which was related to the C = O vibrations of the carboxyl group, indicating the presence of the carboxyl group on the surface of PS [54,55]. The peak at 754 cm^-1^ was attributed to the flexural vibrations (δ C- H) of the benzene ring [56]. The peak observed at 3439 cm^-1^ corresponded to the O-H bond in water molecules [57]. The results confirmed the successful synthesis of carboxyl-functionalized PS microspheres. Spectrum b shows additional peaks at 590 cm^-1^, indicating the stretching vibrations of the Fe-O bond and the integration of Fe_3_O_4_ nanoparticles into the structure [58]. After adding the SiO_2_ layer, a strong peak appeared at 1095 cm^-1^ in spectrum c. This peak resulted from the stretching vibration of the Si-O-Si bond and indicated the existence of a SiO_2_ layer, confirming the presence of the SiO_2_ coating on the PS-Fe_3_O_4_ microspheres [59]. Spectrum d did not contain any PS peaks. Comparison of spectra c and d revealed that high-temperature calcination successfully removed all PS from the structure. Spectrum e showed new peaks at 1455 cm^-1^ and 1639 cm^-1^, which correspond to the C-O and N-H bonds in chitosan. This indicated that chitosan was effectively bonded to the hollow Fe_3_O_4_-SiO_2_ surface [60]. After the adsorption of As(V) and MO, the –OH and –NH₂ peaks of chitosan at 3431 cm ⁻ ¹ and the C– O peak at 1455 cm ⁻ ¹ showed reduced intensity and shifted to lower wavenumbers, while the N–H band at 1639 cm ⁻ ¹ shifted to a higher wavenumber. This could indicate the involvement of these groups in the adsorption process [53,61]. A comparison of the spectra before and after MO adsorption shows an augmentation in the peak areas attributed to the – CH_3_ and – CH_2_ groups (2926 and 2857 cm^−1^). Also, the characteristic stretching vibrations associated with MO, such as C = C (1672 cm^−1^) and those related to the aromatic ring (1551, 643−765 cm^−1^) and stretching vibration of S = O group at 1117 cm^-1^, were observed following adsorption, while they were not present in the spectrum before adsorption. In addition, the peaks observed at 1409–1488 cm ⁻ ¹ can be attributed to the stretching vibration of the azo (–N = N–) group in MO. All these changes confirmed successful adsorption of MO onto the Hollow Fe_3_O_4_-SiO_2_-chitosan [61,62]. After adsorption of As(V), a new peak at 706 cm^-1^ appeared in spectrum G, which can be attributed to the AS-O stretching vibration in the arsenate ion [53]. The changes of the O–H or N–H bands are attributed to the hydrogen bonding interactions between the functional groups in MO and As(V) and the O-containing or N-containing groups (-OH, -NH_2_) on the surface of Hollow Fe_3_O_4_-SiO_2_-Chitosan. Moreover, the shifting of the N-H bond can be related to the electrostatic interaction between the –SO_3_Na group of MO and the As(V) anion with the –NH_2_ functional group on the chitosan surface. The reduction in the intensity of the C–O stretching vibration and the shift of its peak after methyl orange adsorption likely result from the adsorption of the methyl orange molecule onto the adsorbent via n–π interactions [53,61,63]. The Fe–O peak remained unchanged after the adsorption of As(V) and MO, indicating that this bond did not participate in the adsorption of arsenate and methyl orange.

Fig 2B displays the XRD patterns of the adsorbent at different stages of its synthesis. Pattern a showed a broad peak at 19.54°, indicating the amorphous structure of PS [64]. The diffraction patterns visible at 30° (220), 35° (311), 43° (400), 53° (422), 57° (511), and 63° (440) in pattern b showed the spinel cubic crystal structure of Fe_3_O_4_ (JCPDS card no. 19–0629), and confirmed its existence in the PS-Fe_3_O_4_ composites [56]. The peak around 20° in pattern c was attributed to the formation of the amorphous SiO_2_ on the surface of the composite, which showed the coating of the Fe_3_O_4_ nanoparticles with SiO_2_ [65]. After the PS was removed from the structure, pattern d showed the same results as the previous step, indicating that the crystal structures of Fe_3_O_4_ were preserved after calcination at high temperatures. The peak observed at 2θ = 20.52° in pattern e is attributed to the presence of chitosan in the final structure [66].

**Fig 3:**
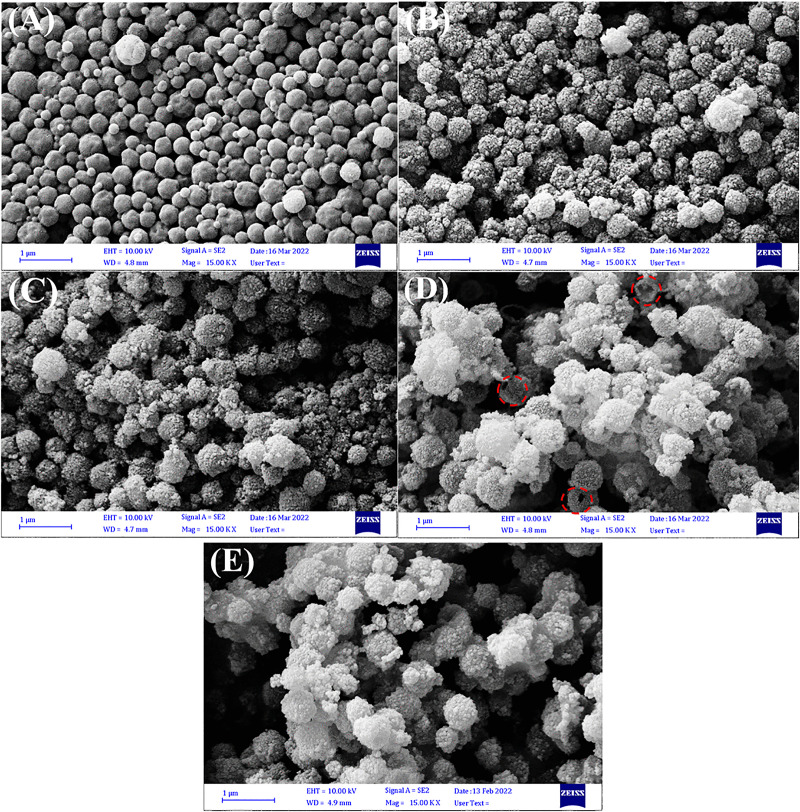
The FESEM images of (A) PS, (B) PS-Fe_3_O_4_, (C) PS-Fe_3_O_4_-SiO_2_, (D) hollow Fe_3_O_4_ SiO_2_, (E) hollow Fe_3_O_4_-SiO_2_-chitosan microspheres.

Fig 3 shows the surface morphology and relative shape of the synthesized materials using FESEM analysis at different synthesis stages. The PS microspheres exhibited a spherical morphology. The variation in PS microspheres sizes is due to secondary nucleation in the polymerization process. As can be seen, the surface of microspheres does not look smooth, which is probably due to the swelling of the structure of microspheres during the drying process of adsorbed water related to the hydrophilic COOH functional groups (Fig 3A) [67]. In Fig 3B, the adsorbent structure is shown in the presence of Fe_3_O_4_ nanoparticles. The overall structure remained spherical, and Fe_3_O_4_ nanoparticles were bound to the PS surface in a nearly spherical form, which made the surface of the microspheres rougher than that of the PS [68]. From the FESEM image of the PS-Fe_3_O_4_-SiO_2_ sample (Fig 3C), a smoother morphology can be observed [69]. As observed in Fig 3D, maintaining the spherical morphology of most of the Fe_3_O_4_-SiO_2_ microspheres after calcination at high temperatures indicates their good thermal stability. Also, the hollow structure formed after calcination can be seen in occasionally broken microspheres [70]. Finally, no significant changes were observed in the hollow Fe_3_O_4_-SiO_2_-chitosan; only the accumulation of particles around this sample seems to be a little more than in the previous example, which indicates the presence of chitosan on its surface. (Fig 3E) [71].

**Fig 4:**
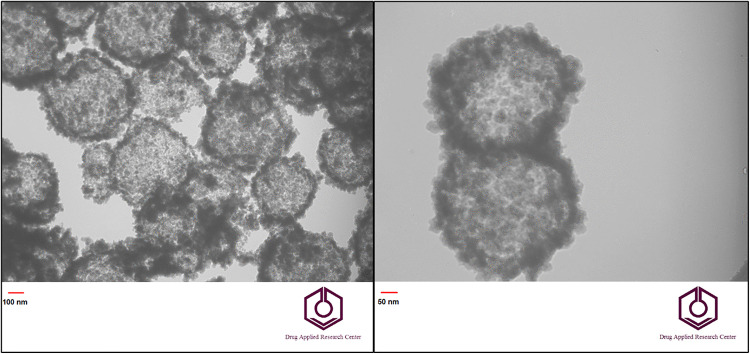
TEM images of hollow Fe_3_O_4_-SiO_2_-chitosan microspheres.

TEM analysis was used for a more detailed study of the Fe_3_O_4_-SiO_2_-chitosan sample core-shell structure. The obtained results are shown in Fig 4. The interesting and unique structure observed indicates the adsorbent had a hollow and porous structure with black spots of magnetic Fe_3_O_4_ nanoparticles and a surrounding gray SiO_2_ layer.

**Fig 5:**
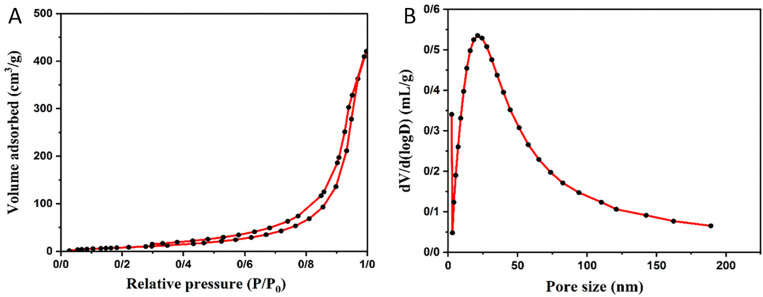
(A) N_2_ adsorption-desorption isotherms, (B) pore size distributions of hollow Fe_3_O_4_-SiO_2_-chitosan microspheres.

The surface area and pore size distribution of the final adsorbent were measured using the BET and BJH methods. Fig 5A shows the nitrogen adsorption-desorption isotherm of hollow Fe_3_O_4_-SiO2-chitosan. These isotherm curves were obtained at 77° K. According to IUPAC, the adsorbent exhibited type IV isotherm with an H3 hysteresis loop, indicating a mesoporous material [72,73]. The results showed that hollow Fe_3_O_4_ -SiO_2_-chitosan had a specific surface area of 202.174 m^2^/gr. Furthermore, the final adsorbent had a total pore volume of 0.653 cm^3^/g and an average pore diameter of 12.91 nm. Fig 5B shows that the adsorbent structure had a range of pore sizes, including micropores, mesopores, and macropores. The highest peak of the graph at around 20 nm confirmed that a significant number of mesopores were present in the adsorbent structure. A wide distribution range of connected pores could improve liquid flow and lead to exceptional adsorption performance of the adsorbent. The specific surface area value indicates that the prepared adsorbent is suitable for adsorption processes [72,74].

**Fig 6:**
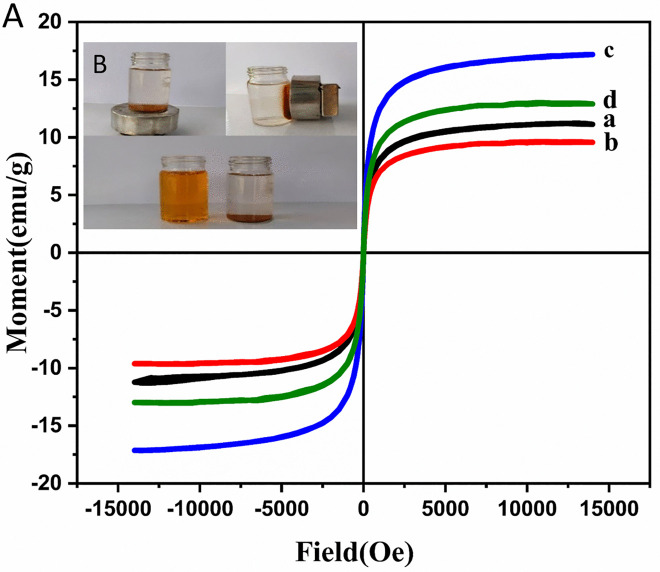
(A) The magnetic hysteresis loops of (a) PS-Fe_3_O_4_, (b) PS-Fe_3_O_4_-SiO_2_, (c) hollow Fe_3_O_4_- SiO_2_, (d) hollow Fe_3_O_4_-SiO_2_-chitosan microspheres, (B) Separation of hollow Fe_3_O_4_-SiO_2_-chitosan microspheres from aqueous medium using an external magnet.

Fig 6A shows the magnetic hysteresis curves at 25°C to evaluate the magnetic properties of the synthesized microspheres. The saturation magnetization values of PS-Fe_3_O_4_, PS-Fe_3_O_4_-SiO_2_, hollow Fe_3_O_4_-SiO_2_ and hollow Fe_3_O_4_-SiO_2_-chitosan microspheres were 11.22 emu/g, 9.64 emu/g, 17.17 emu/g, and 13.00 emu/g, respectively. The reduced saturation magnetization of PS-Fe_3_O_4_-SiO_2_, compared to PS-Fe_3_O_4_, was due to the presence of the nonmagnetic SiO_2_ [60]. The saturation magnetization of hollow Fe_3_O_4_-SiO_2_ was higher than PS-Fe_3_O_4_ and PS-Fe_3_O_4_-SiO_2_ because the mass ratio of Fe_3_O_4_ increased after the calcination process and degradation of PS at high temperatures [75]. Furthermore, the saturation magnetization of the final structure decreased after incorporating chitosan compared to the hollow Fe_3_O_4_-SiO_2_ sample. This reduction can be attributed to introducing a new compound into the structure and reducing the mass ratio of Fe_3_O_4_ magnetic nanoparticles to the total composite [76]. The magnetic separation ability of the adsorbent in solution was evaluated using an external magnet. The adsorbent particles were immediately attracted to the magnet and finally collected by it within seconds. Therefore, according to the results, it can be said that the synthesized magnetic cores provided a practical and effective ability to separate the hollow Fe_3_O_4_-SiO_2_-chitosan adsorbent from an aqueous solution under the influence of an external magnetic field. Fig 6B shows the magnetic response of the adsorbent in the solution when exposed to an external magnetic field.

### 3.2. Adsorption performance

**Fig 7:**
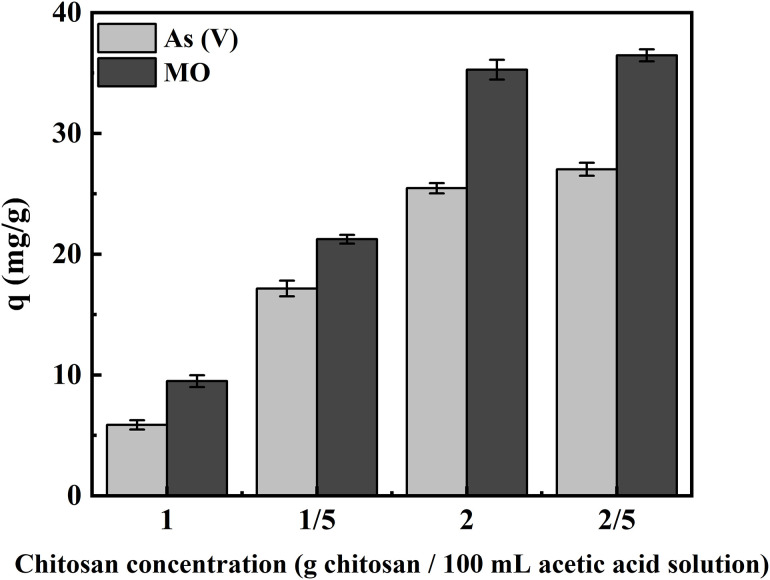
Effect of chitosan concentration on As(V) and MO adsorption capacity.

Fig 7 displays the effects of different weight/volume percentages (g of chitosan/100 mL of acetic acid solution) of chitosan on the adsorption capacity of As(V) and MO. Increasing the chitosan concentration from 1% to 2.5% (w/v) increased both As(V) and MO adsorption capacity. The increase in adsorption capacity was related to more active sites on the surface of the adsorbent, which resulted from using higher amounts of chitosan, especially up to 2% (w/v). At concentrations above 2, there was no significant change in adsorption capacity, which could be because the adsorbent was saturated with chitosan. The excessive chitosan coating on the surface caused overcrowding, restricting access of pollutants to the hollow structure. As a result, although the number of active sites increased, the adsorption capacity showed minimal change. Therefore, we chose the optimal concentration of 2% (w/v) chitosan and then used it to synthesize the adsorbents in the following steps. Similar findings have been reported for other chitosan-based composites, where excessive chitosan blocks internal porous cavities, reduces the specific surface area, and limits further adsorption [77].

**Fig 8:**
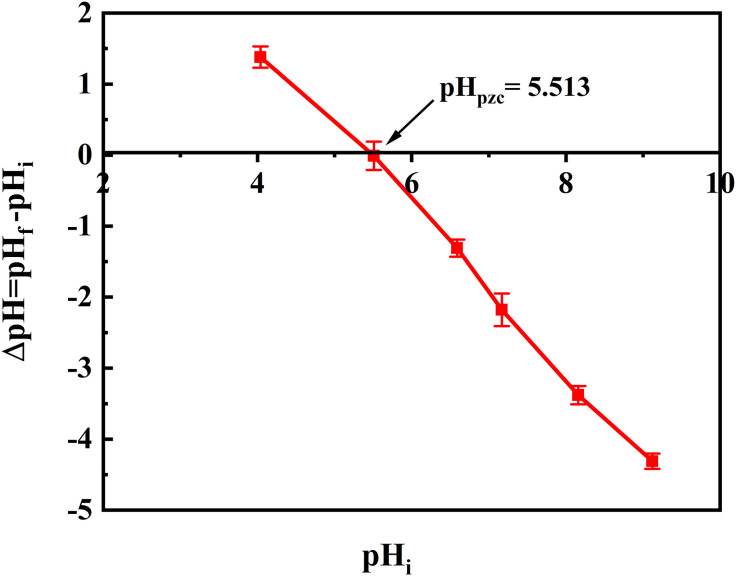
The point of zero charge (pHpzc) of hollow Fe_3_O_4_-SiO_2_-chitosan microspheres.

Fig 8 illustrates the relationship between ΔpH and initial pH for determining the point of zero charge (pHpzc) of hollow Fe_3_O_4_-SiO_2_-chitosan. The pHpzc value of the adsorbent was in the acidic range at pH = 5.513. Electron-rich amino groups of chitosan on the surface of the adsorbent promote the adsorption of positively charged species such as H^+^ in the solution, resulting in a shift of pHpzc to the acidic region [52]. pHpzc can be used to estimate the adsorption behavior of materials at a specific pH value. The adsorbent has a positive surface charge at low pH (pH < pHpzc) due to the protonation of the amino groups in the chitosan and a negative surface charge at pH > pHpzc because of the deprotonation of positively charged amino groups [53].

**Fig 9:**
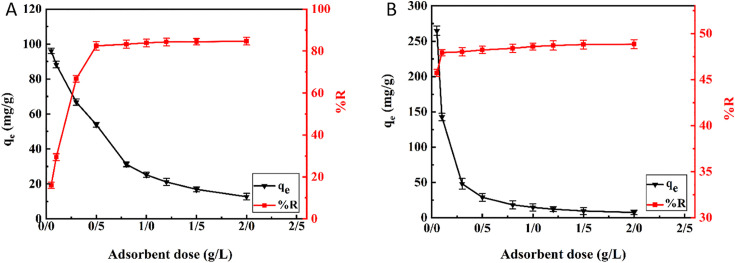
Effect of adsorbent dose on the adsorption capacity of (A) As(V), and (B) MO by hollow Fe_3_O_4_-SiO_2_ chitosan microspheres.

Fig 9 shows how the hollow Fe_3_O_4_-SiO_2_-chitosan dose affects the adsorption capacity and removal efficiency of As(V) and MO. The adsorbent dose was changed from 0.05 to 2 g/L. The results for both pollutants indicated that with an increase in the adsorbent dose, there was an increase in removal efficiency but a decrease in adsorption capacity. The reason is that adding more adsorbent resulted in more available adsorption sites, which increased the removal efficiency of As(V) and MO. On the other hand, adding more adsorbent to the same amount of pollutants resulted in a decreased accessibility of pollutants per unit mass of the adsorbent. This led to some activated adsorption sites remaining unoccupied, and consequently, the adsorption capacity reduced [53]. As the amount of adsorbent rose from 0.05 to 0.5 g/L, the removal efficiency of As(V) increased from 16.021% to 82.512%. However, the removal efficiency remained almost constant at dosages above 0.5 g/L, indicating an equilibrium between adsorbent and As(V) ions. Meanwhile, the adsorption capacity decreased from 96.128 mg/g to 53.873 mg/g. By increasing the adsorbent dose from 0.05 to 0.1 g/L, the removal efficiency for MO improved from 45.720% to 47.929%. The slope of the graph then increased slightly until it reached equilibrium. Furthermore, the adsorption capacity decreased from 264.720 mg/g to 142.544 mg/g. According to Fig 8, 0.5 g/L and 0.1 g/L were considered the ideal and economical dose for As(V) and MO, respectively. The optimal adsorbent dose for MO dye is less than that for As(V) due to differences in their interactions with the adsorbent surface. The As(V) ions are mainly bound to the accessible protonated amine and hydroxyl group in chitosan. This indicates that the As(V) adsorption process mainly depends on the electrostatic interaction between As(V) anions (H_2_AsO_4_^-^) and positive active sites on the adsorbent surface [78]. This explains why the removal efficiency of arsenic improves significantly with a slight increase in the adsorbent dose (Fig 9A). Since the pH of the solution (5) is close the pH pzc of the adsorbent (5.51), the protonation degree of chitosan has begun to decrease and the positive surface charge of adsorbent surface decrease gradually, leading to intense competition between As(V) anions for the available positive sites [13]. Therefore, As(V) requires more adsorbent to have enough active sites for effective removal. While in the adsorption of MO, in addition to electrostatic interactions (between positively charged amino and hydroxyl groups of chitosan and the negatively charged sulfonate groups of MO), hydrogen bonds (between the free H of the hydroxyl group in chitosan and the O, S and N atoms of the MO) may also play a role and n-π interactions could possibly occur as a result of the displacement of lone pair electrons (from the O and N atoms) in chitosan with the π orbitals of the aromatic ring of the MO [79,80]. Therefore, a smaller amount of adsorbent may be enough to remove MO effectively.

**Fig 10:**
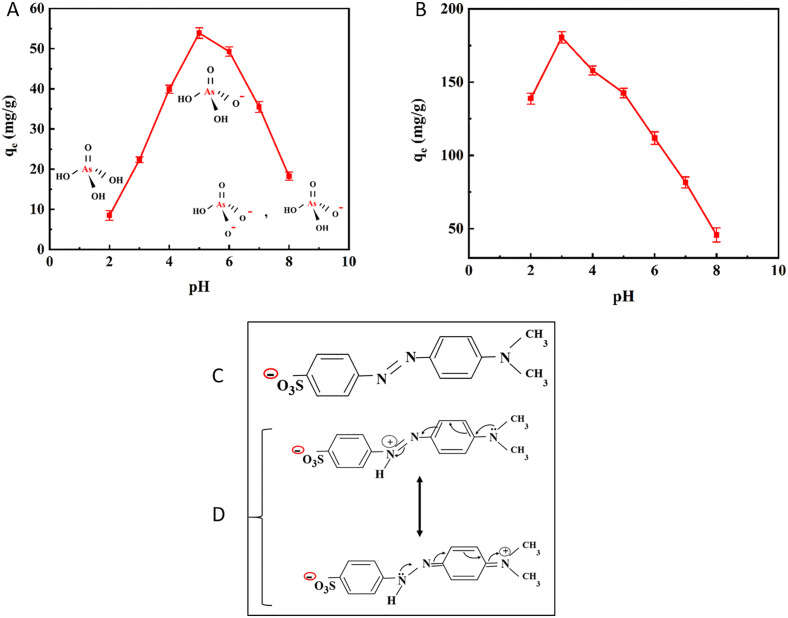
Effect of pH value on the adsorption capacity of (A) As(V), and (B) MO by hollow Fe_3_O_4_-SiO_2_-chitosan microspheres, and molecular structures of anionic MO (C), and zwitterionic forms of MO (D) [83].

pH is an essential factor in adsorption-based water treatment systems. It not only influences the surface charge of the adsorbent but also impacts the speciation of pollutants [81]. Fig 10 shows the effect of pH in the range of 2–8 on As(V) and MO adsorption. At lower pH, H_3_AsO_4_ is the dominant specie of As(V), while in the pH range of 3^_^6, it exists as H_2_AsO_4_^-^, and in the pH range of 6–8 it exists as HAsO_4_^2-^ and H_2_AsO_4_^-^ [13]. Fig 10A shows a two-step variation of As(V) adsorption capacity with pH changes. As the pH value rose from 2 to 3, the adsorption capacity initially increased due to the electrostatic interaction between the As(V) anions and the adsorbent with a positive surface charge (pH < pH_pzc_ = 5.513). In fact, increasing the pH from 2 to 3 changes the dominant As(V) species from H_3_AsO_4_ to a negatively charged H_2_ASO_4_^-^. Subsequently, the adsorption capacity increased up to pH 5 due to the increasing H_2_ASO_4_^-^ species in the solution. In the next step, there was a reduction in the adsorption capacity with an increase in pH from 5 to 8. By increasing pH (pH > pH_pzc_ = 5.513), more negatively charged sites appeared on the adsorbent surface, which decreased As(V) adsorption due to the repulsion effect. Wang et al. [82] reported similar experimental results in the adsorption of As(V) using magnetic nanoparticles-impregnated chitosan beads. For MO, Fig 10B shows that the adsorption capacity decreased with increasing pH from 3 to 8. This is explained by the interaction between the negative sulfonate groups in MO and the positive surface of the adsorbent (pH < pH_pzc_) at lower pH. By increasing the pH, the surface of the adsorbent gradually became more negative due to the deprotonation of the amino groups of chitosan, while MO molecules existed in the solution in anionic form (Fig 10C). Thus, the electrostatic repulsion forces between the adsorbent and MO gradually became stronger and reduced the adsorption. There was also a reduction in adsorption capacity below pH 3. This decrease may be caused by the dye protonating into a zwitterionic form (Fig 10D), which is less favorable for adsorption, or by chitosan dissolving in low-pH environments [83]. Wang et al. [84] observed similar pH trends in the adsorption of MO by carboxyl-functionalized chitosan composite. Therefore, pH 5 and 3 were considered ideal for As(V) and MO, respectively, for the following adsorption experiments.

### 3.3. Adsorption isotherm

**Fig 11:**
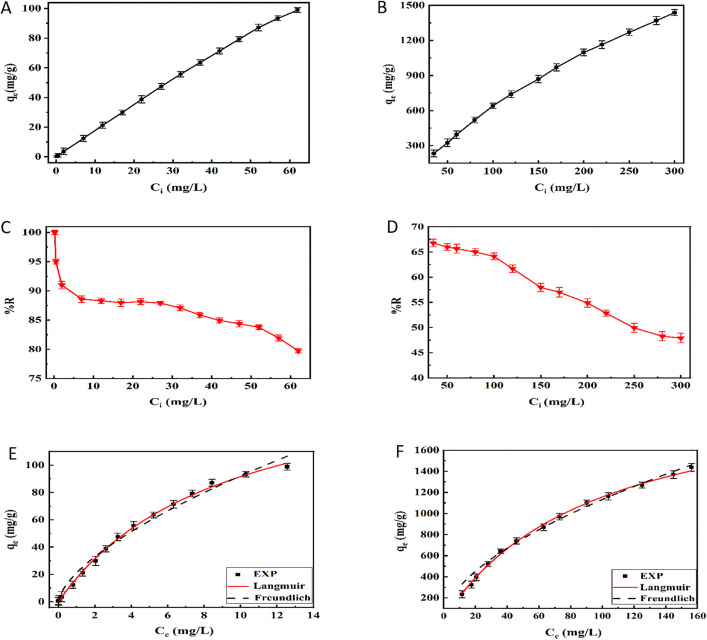
Effect of initial As(V), and MO concentrations on the adsorption capacity ((A) and (B)) and removal efficiency ((C) and (D)) of hollow Fe_3_O_4_-SiO_2_-chitosan microspheres. Langmuir and Freundlich isotherm models of **(E)** As(V), and **(F)** MO, adsorption on hollow Fe_3_O_4_-SiO_2_-chitosan microspheres (pH As **(V)**=5, pH MO = 3, adsorbent dose As(V)=0.5 g/L, adsorbent dose MO = 0.1 g/L, t = 60 min).


Studying the equilibrium isotherm is necessary to understand the adsorption mechanisms of pollutants on the adsorbent surface. In addition, adsorption isotherm models can determine the maximum adsorption capacity, which is essential for assessing the effectiveness of the adsorbents [85]. Fig 11A–11D show the effect of the initial concentrations of As(V) (0.2–62 mg/L) and MO (35–300 mg/L) on the adsorption capacity and removal efficiency of hollow Fe_3_O_4_-SiO_2_-chitosan. The removal efficiency of the adsorbent for both As(V) and MO decreased gradually while the adsorption capacity increased in a straight line as the initial concentration of both pollutants increased, suggesting that the saturation was not achieved in the adsorption process. As the initial concentration rises, the probability of adsorbate collisions on the adsorbent surface increases. The concentration gradient of pollutants between the liquid phase and the adsorption sites, which represents the driving force of the adsorbates towards the active sites, rises with increasing initial concentration, leading to an increase in the adsorption capacity [48]. The present study used the nonlinear form of Langmuir and Freundlich models to investigate the adsorption isotherm models of As(V) and MO on the hollow Fe_3_O_4_-SiO_2_- chitosan and fit the experimental data. The Langmuir isotherm model is grounded on the homogeneous structure of the adsorbent and provides information about the maximum adsorption capacity [86]. Meanwhile, the Freundlich isotherm can study multilayer adsorption processes on heterogonous surface energies [87]. The mathematical expressions of the Langmuir Eq. (3) and Freundlich Eq. (4) models are as follows [79,88]:

$$q_{e}=\frac{q_{m}K_{L}C_{e}}{\text{1}+K_{L}C_{e}}$$ (3)

$$q_{e}=K_{F}{C_{e}}^{\frac{\text{1}}{n}}$$ (4)

The variable q_e_ (mg/g) represents the adsorption capacity of As(V) and MO at equilibrium, while C_e_ (mg/L) denotes the equilibrium concentration of As(V) and MO. q_m_ (mg/g) is the maximum adsorption capacity estimated by the Langmuir model, and K_L_ (L/mg) is the Langmuir model constant. In addition, K_F_ ((mg/g)(L/mg)^1/n^) is the Freundlich constant, and n is a dimensionless constant indicating the adsorption intensity. Fig 11E and 11F show the nonlinear curves of the equilibrium models. Also, Table 1 contains the parameters of the equilibrium models. The correlation coefficient (R^2^) values showed strong correlations between the Langmuir isotherm and the obtained experimental data for both pollutants. These results showed that the surface of the adsorbent is homogenous, and the energy of each adsorption site is equal [85]. The adsorption of As(V) and MO dye occurred in a monolayer arrangement on the surface of hollow Fe_3_O_4_-SiO_2_-chitosan. In this model, the maximum adsorption capacities for As(V) and MO were estimated 175.086 (mg/g) and 2399.910 (mg/g), respectively. Song et al. [48] studied the adsorption of As(V) on chitosan-based composite microparticles. Their investigation revealed that the Langmuir isotherm had better fit the experimental data. Also, according to the results of the study by Chen et al. [89], which investigated the adsorption of MO using magnetic chitosan composite, the experimental data were more consistent with the Langmuir model.

**Table 1:** Adsorption isotherm parameters of As(V) and MO on hollow Fe_3_O_4_-SiO_2_-chitosan microspheres.

	Langmuir isotherm			Freundlich isotherm		
	q_m_ (mg/g)	K_L_ (L/mg)	R^2^	n	K_F_ (L/g)	R^2^
As(V)	175.086	0.109	0.998	1.566	21.205	0.986
MO	2399.910	0.0093	0.992	1.583	61.002	0.976

### 3.4. Adsorption kinetic

**Fig 12:**
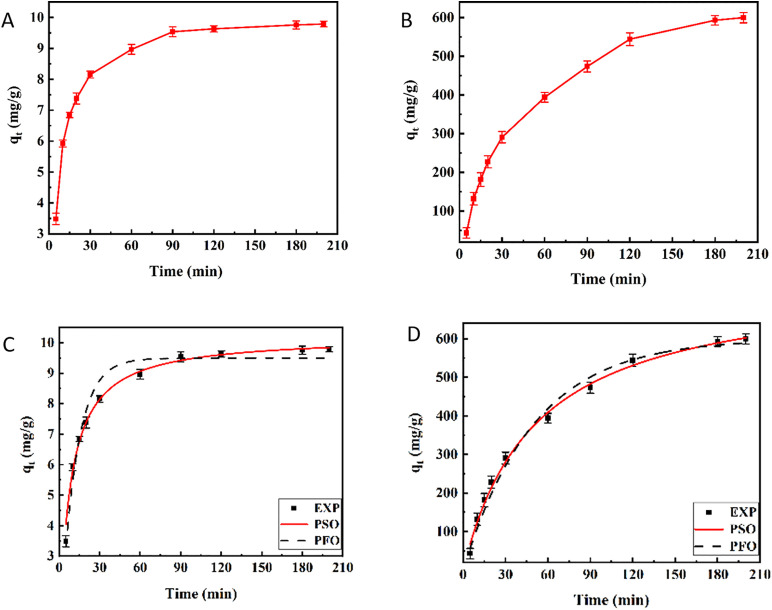
Effect of contact time on (A) As(V), and (B) MO adsorption capacity by hollow Fe_3_O_4_-SiO_2_-chitosan microspheres. Pseudo-first kinetic and pseudo-second kinetic models of **(C)** As(V), and **(D)** MO adsorption on hollow Fe3O4-SiO2-chitosan microspheres (pH As **(V)**=5, pH MO = 3, adsorbent dose As(V)=0.5 g/L, adsorbent dose MO = 0.1 g/L, Ci As(V)= 5 mg/L, Ci MO = 60 mg/ **L)**.


In order to comprehend the control mechanism of the adsorption process of As(V) and MO dye on the adsorbent, a kinetic study was carried out. Fig 12A and 12B show the impact of contact time (5–200 min) on the adsorption capacity of As(V) and MO. The adsorption of both pollutants increased by increasing contact time until it reached equilibrium after 90 min for As(V) and after 180 min for MO. The initial increase in the adsorption capacity is related to the presence of many active adsorption sites on the surface of the adsorbent at the beginning of the adsorption process. However, over time, The adsorbent surface became fully saturated with the adsorbate, leading to the eventual establishment of dynamic equilibrium [84]. In this study, nonlinear form of pseudo-first-order (PFO) and pseudo-second-order (PSO) kinetic models were used to fit the kinetic data. Eq. (5) and Eq. (6) denote the equations of the PFO and PSO models, respectively [90].

$$q_{t}=q_{e}(\text{1}-exp\left({-K}_{\text{1}}t\right))$$ (5)

$$q_{t}=\frac{{q_{e}}^{\text{2}}\;K_{\text{2}}\;t}{\text{1}+q_{e}K_{\text{2}}\;t}$$ (6)

Where qe (mg/g) denotes the adsorption capacity of As(V) and MO at equilibrium, qt (mg/g) is the adsorption capacity of As(V) and MO at time t, K_1_ (1/min) is the rate constant of the PFO model, K_2_ (g/mg.min) is the rate constant of the PSO model, and t (min) represents the time. Fig 12C and 12D show the nonlinear fitting plots of these two kinetic models. Table 2 presents the kinetic parameters derived from the fitting of the experimental data. The obtained R^2^ values confirmed that the adsorption of both pollutants followed the PSO kinetic model. The experimental qe values for As(V) and MO (q_e_^exp^) were closer to the calculated values (q_e_^cal^) obtained from the PSO kinetic models. The results were consistent with previous studies. Ayub et al. [53] investigated As(V) removal on magnetic chitons biosorbent beads, and the results suggested that PSO kinetics was the most suitable model for understanding the kinetics of As(V) adsorption. Tanhaei et al. [91] studied the adsorption of MO using a chitosan-Al_2_O_3_-magnetite nanoparticles composite. They found that the kinetic data fit the PSO model better according to the R^2^ values.

**Table 2:** Adsorption kinetics parameters of As(V) and MO on hollow Fe_3_O_4_-SiO_2_-chitosan microspheres.

	q_e_^exp^ (mg/g)	PFO			PSO		
		q_e_^cal^ (mg/g)	K_1_	R^2^	q_e_^cal^ (mg/g)	K_2_	R^2^
As (V)	9.798	9.495	0.0856	0.954	10.223	0.0127	0.992
MO	599.966	595.213	0.0199	0.988	755.229	0.0002	0.995

### 3.5. Adsorption thermodynamics

The thermodynamics of adsorption were investigated by calculating the standard Gibbs free energy (ΔG°), standard enthalpy (ΔH°), and standard entropy (ΔS°) changes using the following equations [92]:


$$\Delta G˚=-RTln\;K_{C}$$ (7)

$$K_{c}=\frac{C_{a}}{C_{e}}$$ (8)

Here, K_c_ represents the thermodynamic equilibrium constant; R is the universal gas constant (8.314 J/mol·˚K), and T is the absolute temperature (˚K). C_a_ and C_e_ refer to the equilibrium concentrations (mg/L) of the pollutants on the adsorbent surface and in the solution, respectively. The Gibbs free energy (ΔG°) can also be related to ΔH° and ΔS° through the Van’t Hoff equation:


$$-\frac{\Delta G˚}{RT}=\text{ln}\left(K_{C}\right)=\frac{-\Delta H˚}{RT}+\frac{\Delta S}{R}$$ (9)

ΔH° and ΔS° were determined from the slope and intercept of the ln (Kc) vs. 1/T plot. The thermodynamic analysis was conducted at three different temperatures (25, 35, and 45°C) and the results are presented in Table 3. The negative ΔG° values at different temperatures indicate that the adsorption process is spontaneous and thermodynamically feasible with minimal energy input from the environment. Moreover, the increasing negativity of ΔG° with rising temperature suggests that the adsorption of As(V) and MO onto Hollow Fe_3_O_4_-SiO_2_-chitosan becomes more favorable at higher temperatures. The positive ΔH° values imply that the adsorption is endothermic for both MO and As(V), explaining the enhancement of adsorption capacity with increasing temperature. Additionally, the positive ΔS° values indicate an increase in randomness at the solid/solution interface, reflecting greater mobility of As(V) and MO ions during the adsorption process. Similar results regarding the removal of methyl orange dye and arsenate ions using chitosan-based adsorbents have been reported in previous studies [62,93].

**Table 3:** Thermodynamic adsorption parameters for As(V) and MO adsorption by hollow Fe_3_O_4_-SiO_2_-chitosan.

	T(˚K)	ΔG˚ (KJ/mol)	ΔH˚ (KJ/mol)	ΔS˚ (KJ/mol.K)
As(V)	298.15	−4.927	57.822	0.210
	308.15	−7.437	–	–
	318.15	−9.136	–	–
MO	298.15	−1.179	12.100	0.044
	308.15	−1.760	–	–
	318.15	−2.063	–	–

### 3.6. Effect of coexisting anions

**Fig 13:**
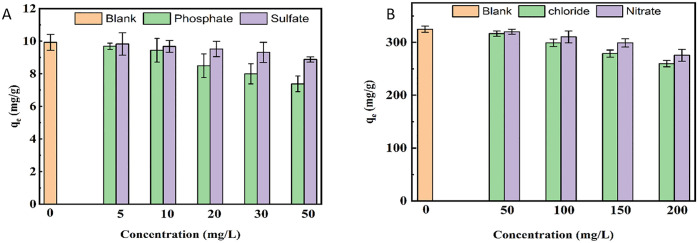
Effect of coexisting ions concentration on the adsorption of (A) As(v) and (B) MO by hollow Fe_3_O_4_- SiO_2_-chitosan (pH As (V)=5, pH MO = 3, adsorbent dose As(V)=0.5 g/L, adsorbent dose MO = 0.1 g/L, Ci As(V)= 5 mg/L, Ci MO = 50 mg/ L, contact time As(V) and MO = 60 min).

Since the coexisting ions can compete with As(V) and MO for the available adsorption sites, evaluating the effect of ionic strength on the adsorption behavior is essential. Fig 13 illustrates the effect of competing phosphate and sulfate ions on the adsorption of As(V), as well as the influence of competing chloride and nitrate ions on the adsorption of MO. As shown in Fig 13A, the adsorption capacity of As(V) was reduced in the presence of competing ions. Phosphate exerted a stronger competitive effect than sulfate, which can be attributed to its similarity with As(V) in terms of ionic size, structure, and pKa, as well as P and As closely related positions in the periodic table. Due to these similarities, phosphate competes more strongly with As(V) for the same adsorption sites [94]. Nevertheless, the adsorbent was able to preserve a substantial fraction of its initial adsorption capacity, which was obtained in the absence of competing ions, even after the introduction of competing species. Even at elevated concentrations of competing ions (50 mg/L), it retained approximately 74% of the original adsorption capacity in the presence of phosphate and 89% in the presence of sulfate. In the case of MO, as shown in Fig 13B, the adsorption capacity of the adsorbent decreased with increasing concentrations of chloride and nitrate ions. Compared to nitrate, the presence of chloride ions caused a more pronounced reduction in MO adsorption capacity, suggesting that Cl⁻ ions competed more strongly with MO molecules for the available adsorption sites [84,90]. As the chloride concentration increased, this competition became more intense. However, even at high concentrations of chloride and nitrate ions, the adsorbent retained approximately 80% of its initial adsorption capacity in the presence of chloride ions and 84% in the presence of nitrate ions.

### 3.7. Desorption study

**Fig 14:**
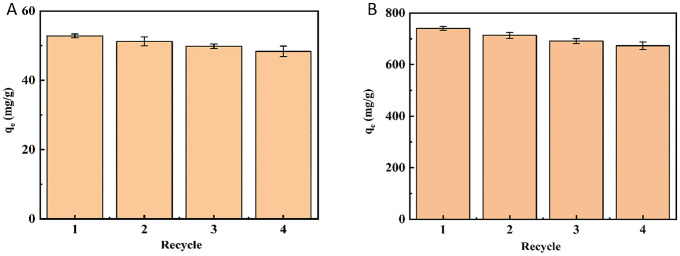
Regeneration of hollow Fe_3_O_4_-SiO_2_-chitosan microspheres for the removal of (A) As(V) and (B) MO.

Regeneration performance plays a crucial role in evaluating the potential application of adsorbents in real systems. As shown in Fig 14, the adsorption capacities for both As(V) and MO exhibited a slight decrease after several reuse cycles, which could be attributed to incomplete desorption and the partial loss of active adsorption sites on the adsorbent. However, the adsorbent retained more than 91% of its initial adsorption capacity for As(V) and over 90% for MO after four consecutive adsorption–desorption cycles. Thus, the hollow Fe₃O₄–SiO₂–chitosan adsorbent exhibits excellent regeneration ability and reusability for the adsorption of MO dye and As(V) ions.

### 3.8. Comparison with the adsorption data from other works

Table 4 compares the maximum adsorption capacity (qm) and adsorption conditions of hollow Fe_3_O_4_-SiO_2_-chitosan in As(V) and MO adsorption with other chitosan adsorbents previously documented in the references. The synthesized microspheres in this work demonstrated noticeably better adsorption performance for As(V) and MO adsorption with the lowest optimal adsorbent dose compared to other works. These results suggest they have the potential to be an effective adsorbent for removing As(V) and MO from aqueous solutions.

**Table 4:** Comparison of hollow Fe_3_O_4_-SiO_2_-chitosan and non-hollow magnetic chitosan adsorbents in As(V) and MO adsorption.

Adsorbent	pH	pollutant	Adsorbent dose (g/L)	q_max_ (mg/g) (Langmuir)	Ref.
Magnetic chitosan-based composite	7.0	As(V)	3.0	34.610	[48]
Magnetic chitosan biosorbent beads	6.7	As(V)	1.5	79.490	[53]
Chitosan-magnetite nanocomposite	7.0	As(V)	1.0	10.810	[95]
Iron chitosan Microspheres	5.2	As(V)	1.0	120.70	[63]
ChitosanMGOP-Co_3_O_4_	7.0	As(V)	1.2	91.00	[96]
Chitosan-CoFe_2_O_4_/BC	4.0	MO	0.5	644.00	[97]
AL-Chitosan-MNPs	5.0	MO	2.0	329.500	[52]
C-Chitosan-ZnO	2.0	MO	1.0	185.200	[98]
Hollow Fe_3_O_4_-SiO_2_-chitosan	5.0	As(V)	0.5	175.086	This work
Hollow Fe_3_O_4_-SiO_2_-chitosan	3.0	MO	0.1	2399.910	This work

## 4. Conclusion

In this study, a hollow Fe_3_O_4_-SiO_2_-chitosan adsorbent with a 2% (w/v) chitosan concentration was synthesized. The unique internal hollow structure, high specific surface area, the selection of the optimal chitosan concentration for synthesis, and minimal adsorbent requirement enhanced its cost-effectiveness while offering adequate and effective adsorption sites for removing arsenic(V) from low-concentration aqueous solutions. Additionally, its adsorption performance was evaluated for methyl orange dye, an organic pollutant. Adsorption data for both contaminants were well fitted to the Langmuir isotherm and the pseudo-second-order kinetic model, confirming monolayer adsorption and chemisorption behavior. The maximum adsorption capacities were calculated as 175.086 mg/g for arsenic(V) and 2399.910 mg/g for methyl orange, significantly surpassing similar adsorbents. A key finding of this study was the high removal efficiency (100%) in removing arsenic(V) at a low concentration of 0.2 mg/L using a lower adsorbent dose of 0.5 g/L compared to previous studies, demonstrating its economic and practical advantages. Furthermore, optimizing the chitosan concentration was critical to maximizing pollutant-adsorbent interactions. While increasing the chitosan concentration enhanced the number of active adsorption sites, it did not significantly improve overall adsorption capacity. This was attributed to surface saturation at higher chitosan concentrations, which hindered access of the pollutants to the interior of the adsorbent. Also, the adsorbent demonstrated strong selectivity, maintaining a substantial fraction of its original adsorption capacity for both As(V) and methyl orange, even in the presence of competing anions such as phosphate, sulfate, chloride, and nitrate. Furthermore, after four adsorption–desorption cycles, it retained over 90% of its adsorption capacity, highlighting its potential for repeated use and practical applications in wastewater treatment. In conclusion, the hollow magnetic Fe_3_O_4_-SiO_2_-chitosan adsorbent, with its high adsorption capacity, rapid separation ability, and cost-effectiveness, emerges as a promising candidate for the removal of both methyl orange and arsenic(V) pollutants in practical applications.

## Supporting information

S1 Data Adsorbent dose. (XLSX)

S2 Data As(v) Competition. (XLSX)

S3 Data BET. (XLSX)

S4 Data Concentration of chitosan. (XLSX)

S5 Data Desorption. (XLSX)

S6 Data FTIR. (XLSX)

S7 Data Isotherm. (XLSX)

S8 Data Kinetic. (XLSX)

S9 Data Methyl orange competition. (XLSX)

S10 Data pH. (XLSX)

S11 Data Thermodynamic. (XLSX)

S12 Data VSM. (XLSX)

S13 Data XRD. (XLSX)
